# Prospective Study of Aetiopathogenesis and Monitoring of Intra-abdominal Pressure for Early Detection of Burst Abdomen

**DOI:** 10.7759/cureus.77415

**Published:** 2025-01-14

**Authors:** Gulab Dhar Yadav, Anju Yadav, Gajendra Pratap Singh

**Affiliations:** 1 Department of General Surgery, Ganesh Shankar Vidyarthi Memorial Medical College, Kanpur, IND; 2 Department of Pediatrics, Employees State Insurance Corporation Hospital, Kanpur, IND

**Keywords:** elective laparotomy, wound dehiscence, burst abdomen, emergency laparotomy, intraabdominal pressure, risk-factors

## Abstract

Background: The risk of evisceration, the need for rapid treatment, and the possibility of recurrent dehiscence make burst abdomen a severe postoperative complication that surgeons must deal with. A wound's dehiscence is linked to severe morbidity and mortality. This study examined how rapidly abdominal burst and wound dehiscence could be identified in patients after both emergency and scheduled laparotomies.

Methods: This prospective study included 80 patients with burst abdomens, aged more than 18, who underwent exploratory laparotomies in the Department of General Surgery, LLR, and associated hospitals, Ganesh Shankar Vidyarthi Memorial (GSVM) Medical College from January 2021 to October 2022. Various factors were observed, such as postoperative wound infection, nutritional status, raised intra-abdominal pressure, type of pathology, and patients undergoing emergency or elective exploratory laparotomy.

Results: The presence of intra-abdominal sepsis (63.75%), anemia (61.25%), and hypoproteinemia (50%) favors a higher incidence of burst abdomen as preoperative risk factors. Among various pathologies, gastro-duodenal perforation (30.0%) was found to be the most common pathology associated with a burst abdomen. In the postoperative period, wound infection (77.5.0%) was the most common factor associated with a burst abdomen, followed by raised intra-abdominal pressure (due to cough (35%), vomiting (17.5%)), and postoperative abdominal distension (27.5%). Intra-abdominal pressure was found to be a very sensitive early predictor of wound dehiscence, with peak incidence on POD-6 (IAP>16 mmHg).

Conclusion: Emergency procedures are more likely than elective surgeries to result in a burst abdomen. Anemia and wound infection both raise the risk of abdominal rupture. Intra-abdominal pressure was found to be a very sensitive early predictor of wound dehiscence. In both the treatment and prevention of this disorder, adherence to good methods and serious attempts to reduce the influence of predisposing variables are much more important.

## Introduction

Burst abdomen or wound dehiscence is a significant postoperative complication due to the risks of evisceration, the urgency of treatment, the likelihood of recurrent dehiscence, the development of surgical wound infections, and the potential for incisional hernias. Despite improvements in preoperative and postoperative care over the last several years, wound dehiscence after surgery remains a difficult problem that significantly extends hospital stays and is linked to death rates of 10% to 44% [1]. There is a significant risk of morbidity and death associated with wound dehiscence.

The two most well-known and common consequences in patients with traumatic abdominal injuries who have emergency laparotomy are intra-abdominal sepsis (IAS) and burst abdomen. They are prevalent in 20% of the cases and seriously hinder the outcomes [2]. IAS carries a significant mortality of over 50% if its identification is delayed. It is a precursor to multiple organ failure, abdominal wall defect, acute respiratory distress syndrome, and recurrent hospitalizations to intensive care units (ICU) [3,4].

Some causes of these problems include the scope and severity of the abdominal injury, the increase in intra-abdominal pressure (IAP) during the pre, intra, and post-operative phases, and the operative severity score (OSS). The risk of IAS and burst abdomen increases if the injury is acute or extensive and has a high injury severity score (ISS). The causes include immunosuppression, tissue hypoxia, and widespread hypoperfusion [3]. Raised IAP decreases the renal, mesenteric, pulmonary, and cardiovascular circulation and is a prelude to abdominal compartment syndrome. Bacterial translocation across the gut wall is also likely to happen when the blood supply to the intestinal mucosa decreases [5].

Wound dehiscence cannot be attributed to a single cause; rather, a number of variables are often at play. If the wound's support system fails before its functional and structural integrity is fully restored, the edges of the wound will separate. Several risk factors have been associated with the development of incisional hernias and burst abdomen, including jaundice, anemia, diabetes, COPD, uremia, hypoalbuminemia, steroid use, advanced malignancy, wound infection, obesity, and emergency surgery. However, some of these factors, such as emergency surgery, obesity, jaundice, diabetes, and anemia, have recently been called into question. Among these, wound infection remains the most significant determinant in the occurrence of these conditions [6].

After an emergency laparotomy, a patient's postoperative phase may be dangerous since a burst abdomen might result in a lengthy hospital stay, increased morbidity, or even death. Close monitoring and timely intervention can benefit patients identified as high-risk. Early identification of risk factors significantly reduces the chances of developing a burst abdomen [7].

## Materials and methods

This prospective study was conducted at the Department of General Surgery, LLR, and associated hospitals of Ganesh Shankar Vidyarthi Memorial (GSVM) Medical College on patients who underwent primary surgery and subsequently developed a burst abdomen as a complication between January 2021 and October 2022. Patients of both genders older than 18 years of age, who provided informed permission were included in the study. Before starting the study, the hospital ethics committee's permission was obtained. All subjects provided their informed permission. Taking part in the research was fully voluntary, and the patient had the opportunity to leave at any moment.

Study sample

Patients who had exploratory laparotomies for both emergency and non-emergency abdominal surgeries and experienced postoperative dehiscence throughout the research period were included. Only 80 patients (burst abdomen) were found out of 800 exploratory laparotomies.

Inclusion and exclusion criteria

Patients of any gender over the age of 18 who have signed a permission form for testing and care are included in the study. All patients who were operated primarily in LLR Hospital and developed burst abdomen as complications were included. Patients who had their operations performed predominantly at locations other than LLR Hospital or who previously had laparotomies for whatever reason (or who had an incisional hernia or burst abdomen) were not included in the research.

Data collection

Each case underwent a detailed and systematic clinical examination. The etiological factors analyzed included the patient’s age, sex, indication for surgery, nature of the surgery (emergency or elective), type of incision, duration of surgery, presence of anemia, and the day the burst abdomen occurred. Postoperative wound infection was confirmed using wound swab culture sensitivity. Respiratory infections during the postoperative period were assessed through a history of cough, dyspnea, or both, along with lung auscultation for crepitations and chest X-rays to identify pleural effusion or pneumonitis.

A checkup of the abdomen was done to look for evisceration, wound dehiscence, serosanguinous discharge, infection, and distention. Investigations for blood sugar, wound swabs for culture and sensitivity, serum proteins, hemoglobin, urea, and creatinine levels, and a chest X-ray were performed on all patients with burst abdomens. The etiological variables, examination results, risk factors, and investigations were all included in a thorough proforma. Based on the history, physical, and laboratory research, all risk variables were rendered quantifiable. Patients were thoroughly monitored after surgery.

Method of collection of data

All laparotomies (elective and emergency) were performed using mid-line vertical incisions under general anesthesia. In both emergency and elective situations, antibiotics were started as part of preoperative care for all patients with an acute abdomen. The same operations team was used in every instance. In all cases (elective and emergency), we used en masse closure using a Polydioxanone Suture (PDS) double loop. Each patient had a post-operative examination to check for any wound infections and symptoms of wound dehiscence.

The surgical ward often began inspecting the site on the second postoperative day, noting the presence of any discharge, such as pus or serosanguineous fluid. Following an urgent laparotomy, peritoneal lavage, and drainage, utilizing absorbable suture material, the abdomen's peritoneal layer was sealed. A continuous pattern using a non-absorbable number 1 nylon suture was employed to close the abdominal fascial layers layer by layer. Retention sutures, placed approximately 5 cm apart, were added to reinforce the primary closure and serve as a protective buttress for the skin.

**Figure 1:**
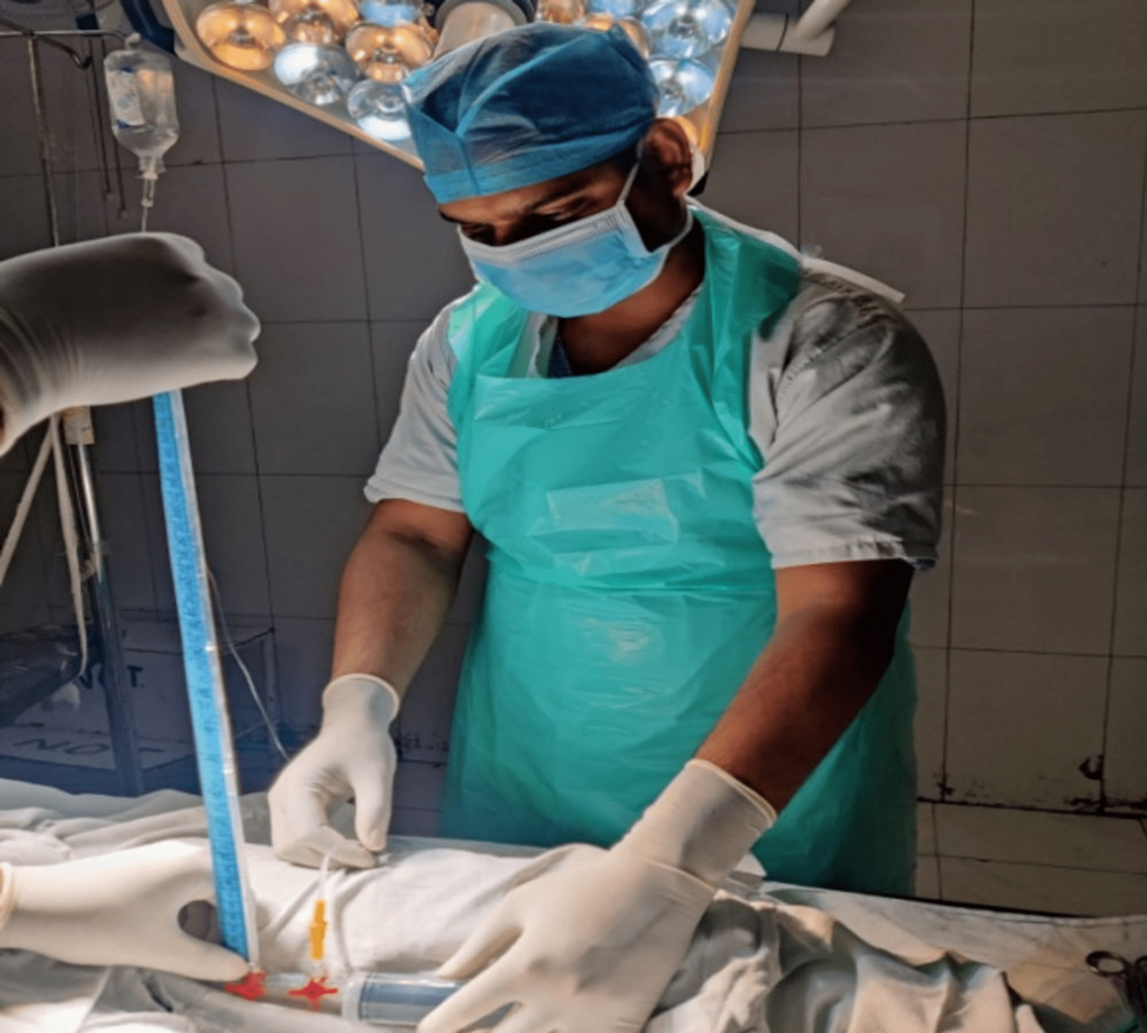
Measurement of intra abdominal pressure using traditional foley's catheterisation method.

**Figure 2:**
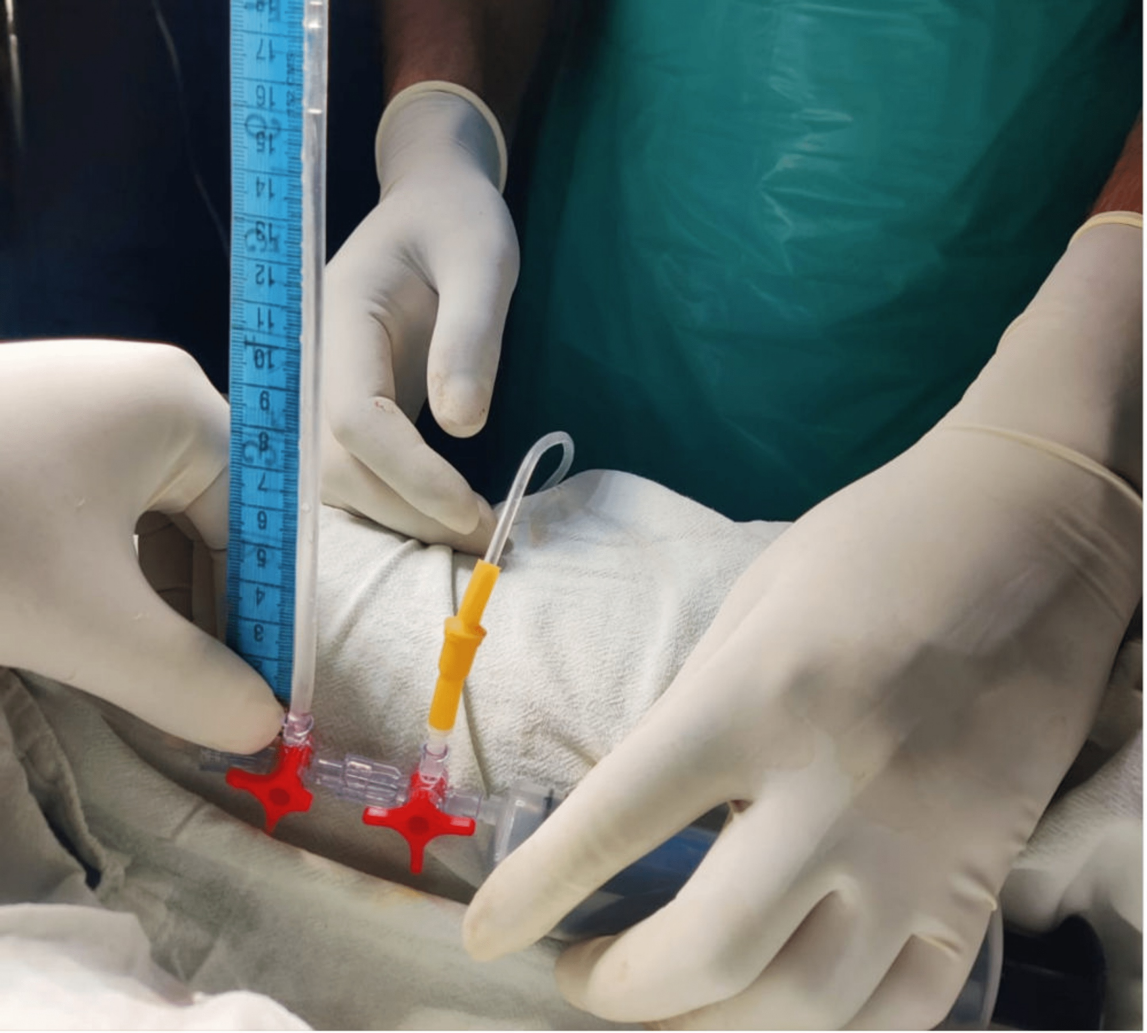
Intra abdominal pressure of 16 cm of water in a post operative patient of exploratory laparotomy.

A drainage bag is connected to the drainage limb of the three-way Foley catheter (urban), while intravenous or pressure tubing and a three-way stopcock are used to attach the water manometer to the catheter's irrigation limb. As shown in the image, a three-way stopcock is attached to a 50 cc syringe filled with saline. The regular saline is used to prime the circuit (heparin or pressurization are not necessary for the flush solution). There is no leftover urine since the catheter is still exposed to continuous drainage before the bladder pressure is measured. The drainage tube was clamped at this point, and then the syringe was used to inject 50 cc of ordinary saline into the bladder. This guarantees that the bladder's fluid capacity remains consistent during each measurement. The manometer was aligned with the bladder, approximately at the level of the pubic symphysis, when the patient was lying supine. The mean pressure measurement was recorded after closing the stopcock on the syringe. Measurements were initially taken in centimeters of water and later converted to millimeters of mercury (mmHg) using the conversion factor 1 mmHg=1.36 cm of water, as per the guidelines of the World Society of the Abdominal Compartment Syndrome (WSACS) (Figure 1 and Figure 2). 

Statistical analysis

The database was created using Microsoft Excel, and graphs were produced using SPSS version 23 for Windows (IBM SPSS Statistics for Windows, Version 23.0), which was also applied to assess the data. Mean, and standard deviation were utilized for continuous variables, while N and percentage values were used for categorical values to define quantitative data meeting normal distribution. Non-normal distribution or continuous factors were compared with Fisher’s exact or Pearson’s Chi-square test, and for means, the student t-test was applied. The significance level was deemed as p<0.05.

## Results

**Figure 3:**
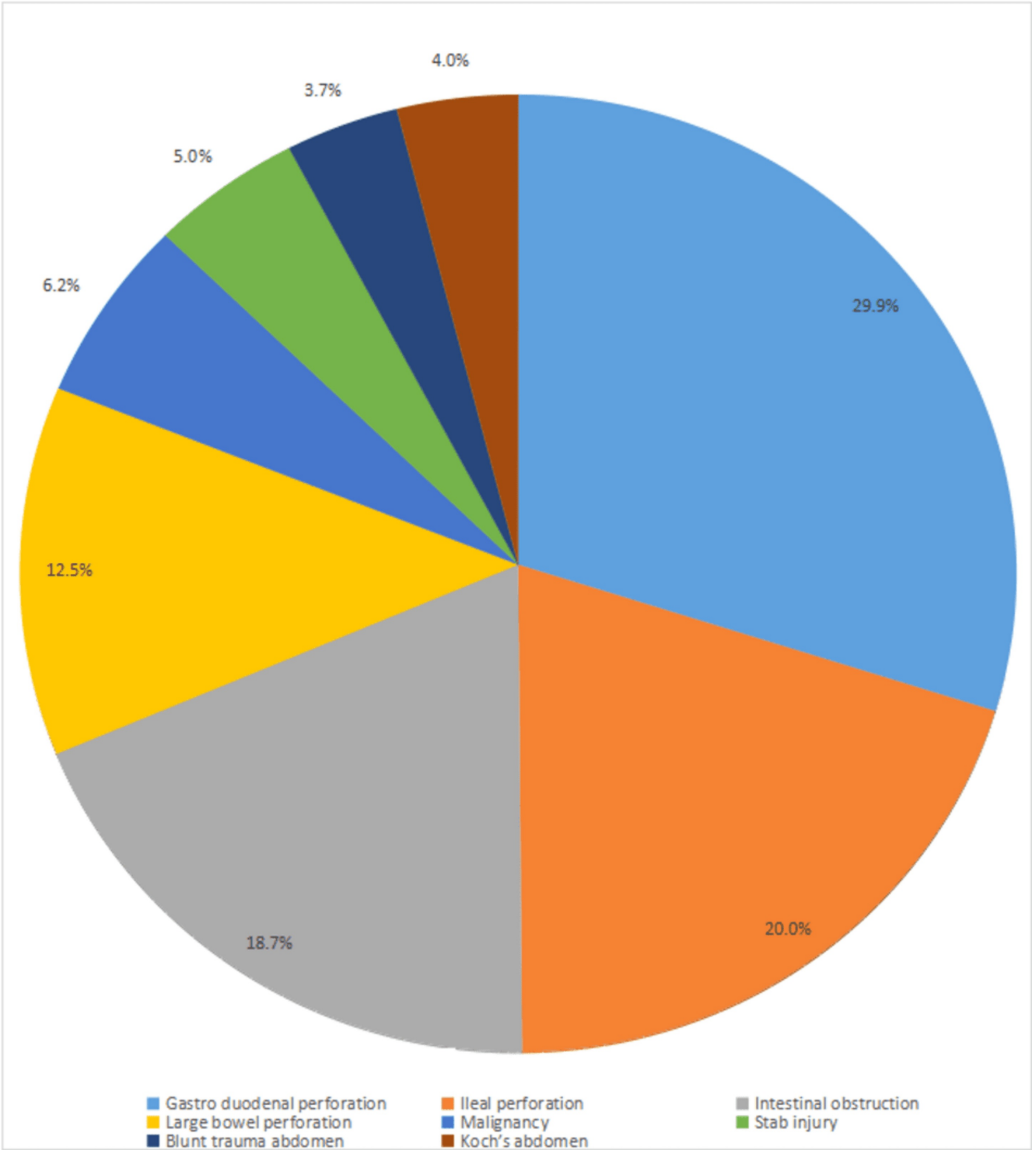
Pathological findings seen in patients intra operatively.

The mean age of the studied cases was 47.25±8.95 years, with male predominance (75.0%). 88.7% of cases underwent elective surgery, whereas 1.3% underwent emergency. The presence of intra-abdominal sepsis (63.75%), anemia (61.25%), and hypoproteinemia (50%) favors a higher incidence of burst abdomen as preoperative risk factors. Pathological findings of subjects were gastro duodenal perforation (20%) which was the most common indicator of bust abdomen followed by ileal perforation (20.0%), intestinal obstruction (18.7%), large bowel perforation (12.5%), malignancy (6.2%) and stab injury (5.0%) (Figure 3). 

**Figure 4:**
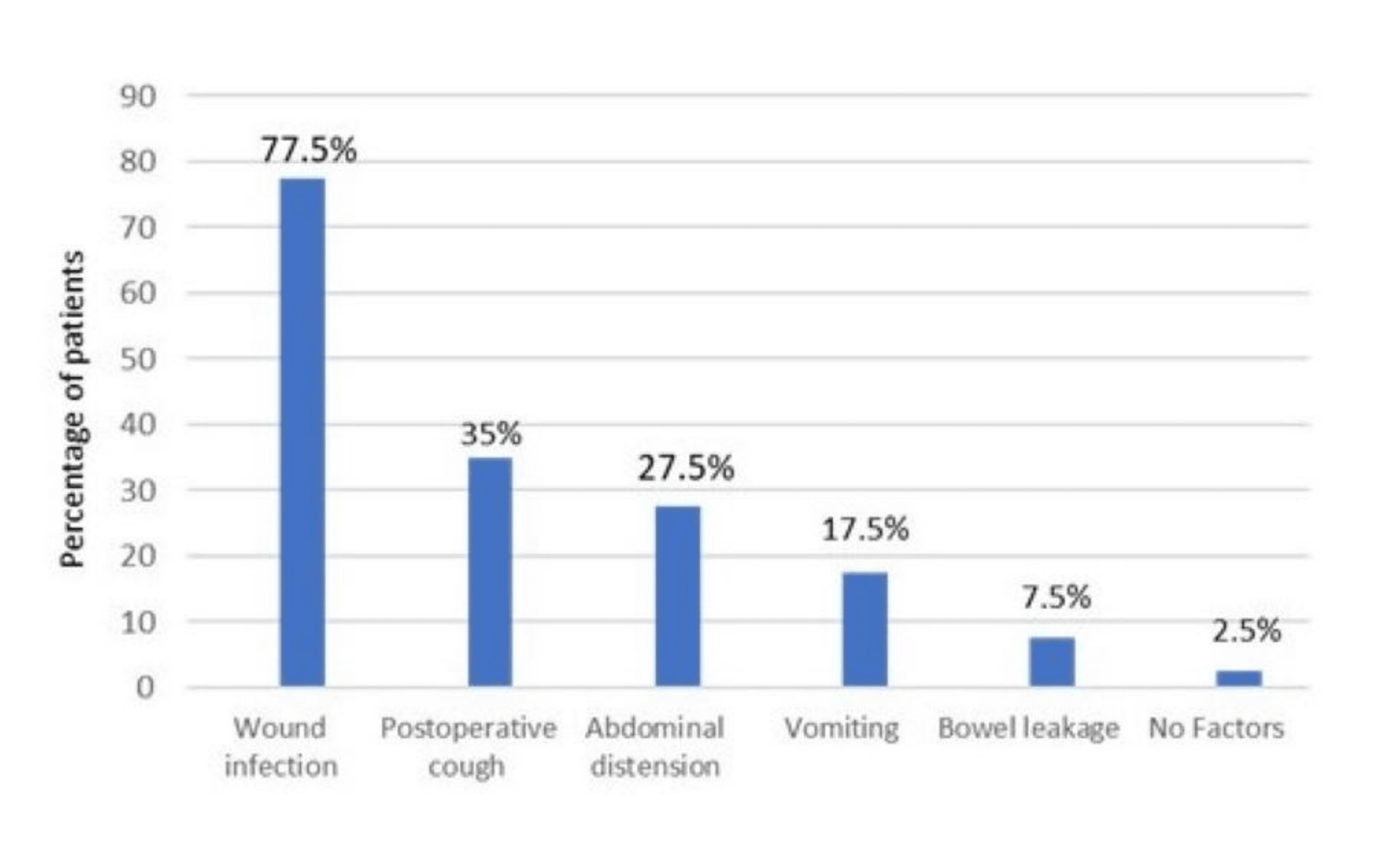
Factors associated in postoperative period.

The most common postoperative complications included wound infection, observed in 77.5% of patients, followed by postoperative cough in 35%, abdominal distension in 27.5%, vomiting in 17.5%, and bowel leakage in 7.5%. Two patients (2.5%) experienced no complications (Table 1, Figure 4).

**Table 1:** Factors associated with burst abdomen.

Complications	No. of cases (n=80)	Percentage
Wound infection	62	77.5 %
Postoperative cough	28	35.0 %
Abdominal distension	22	27.5 %
Vomiting	14	17.5 %
Bowel leakage	6	7.5 %
No Factors	2	2.5 %

**Figure 5:**
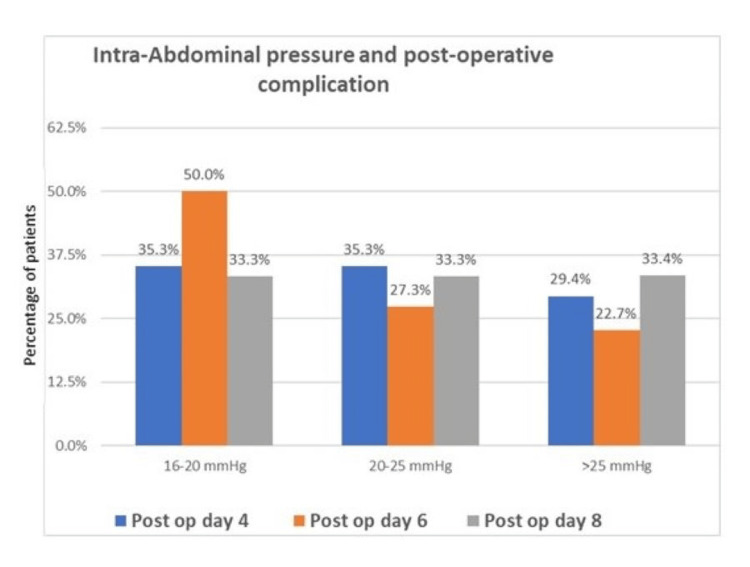
Incidence of burst abdomen on various post operative days at different intra abdominal pressure. Post op day 4: postoperative day 4; Post op day 6: postoperative day 6; Post op day 8: postoperative day 8; IAP: intra-abdominal pressure.

On the basis of sequential intra-abdominal pressure measurement, it was interpreted that peak incidence of raised intra-abdominal pressure was found on postoperative day (POD) 6. Incidence was similar on POD-4 and POD-6 (Table 2, Figure 5).

**Table 2:** Distribution of studied patients who developed burst abdomen as post-operative complication based on intra-abdominal pressure (IAP). This table shows the number of patients developing burst abdomen on different post-operative days with respect to different intra-abdominal pressures.

Intra-abdominal pressure (mmHg)	Incidence of burst abdomen on different postoperative days			p-value
	Day 4	Day 6	Day 8	
16-20 mmHg	6 (35.3%)	11 (50.0%)	1 (33.3%)	0.616
20-25 mmHg	6 (35.3%)	6 (27.3%)	1 (33.3%)	0.862
>25 mmHg	5 (29.4%)	5 (22.7%)	1 (33.4%)	0.857
Total patients	17 (100.0%)	22 (100.0%)	3 (100.0%)	-

## Discussion

Despite advancements in surgical techniques, access to modern technology, and growing clinical expertise, burst abdomens remain a significant contributor to postoperative morbidity and mortality. The risk of developing a burst abdomen depends on factors such as the type of surgery, the patient's underlying overall health, and the underlying condition. The incidence is highest between the sixth and eighth postoperative days. Several factors influence the occurrence of burst abdomens and wound healing, which have been extensively studied. These predisposing factors are typically classified into three categories: general preoperative, local or surgical, and postoperative causes.

Any age group can have a burst abdomen. In this work, the mean age of participants was 47.25±8.95 years with male predominance (75.0%). Our finding was in accordance with the Rashid et al. study, where a maximum of 30% of patients were >50 years of age [7]. Another study by Jaiswal et al. reported similar results, with the mean age of patients being 49 years, and 78% of cases were male in their study [8]. These results are consistent with what Hermosa et al. observed in their series of cases, where they discovered that 24% of the patients were over 50 years old [9].

The most common predisposing factor was intra-abdominal sepsis in 63.75% of subjects, followed by anemia in 61.25% and hypoproteinemia in 50% in our study. Our findings were comparable to the findings of Meena et al. that the occurrence of abdominal wound dehiscence is considerable in peritonitis patients having protracted surgery in the presence of risk factors such as hypoproteinemia, anemia, wound infection, postoperative ileus, and postoperative pulmonary infection [10]. Similarly, Sivender et al. [11] reported that intra-abdominal Infection (IAI) is one of the main risk factors for a burst abdomen since they discovered that IAI was present in 60% of their patients.

According to the current research, peritonitis brought on by a perforation often results in an enlarged abdomen, of whom 30% of cases were due to gastroduodenal perforation. Other IAPs in the current study are intestinal obstruction (18.75%), ileal perforation (20%), Koch’s abdomen (3.75%), malignancy (6.25%), blunt trauma abdomen (3.75%) and stab injury (4%). The most frequent factor contributing to burst abdomen was peritonitis. In the Jaiswal et al. study, 29.26% of cases were due to gastroduodenal perforation [8]. Other IAPs were intestinal obstruction (18.29%), ileal perforation (19.51%), Koch’s abdomen (2.43%), malignancy (14.63%), blunt trauma abdomen (3.65%) and stab injury (4.87%). In some other studies, Halasz et al. [12], Riou et al. [13], and Waqar et al. [14] also reported frequent pathologies of gastro duodenal perforation 25.0%, 12.90%, and 28.57%, respectively.

In the current research, the majority of the studied cases showed wound infection (62.5%) followed by raised intra-abdominal pressure due to post-operative cough (28.75%), abdominal distension (20%), vomiting (13.75%), bowel leakage (6.25%) and no complications (2.5%). Our findings were in accordance with Rashid et al. [7], who noted that wound infection was the most prevalent factor in the post-operative period and that it was present in 62% of instances of burst abdomen, significantly more than the group who did not. Postoperative cough was the second most prevalent contributing cause. Compared to only 4% of group B patients, abdominal distension was seen in 22% of cases with burst abdomen (who didn’t have a burst abdomen). In 14% of instances with burst abdomen, postoperative vomiting was a factor. Afsal et al. [6] in their study reported that 88.75% had wound sepsis, followed by 51.42% having intra-abdominal sepsis, 45.57% bowel fistule, 20.0% pneumonitis, and 5.7% having jaundice.

In our study, sequential measurement of IAP was done (POD-2 to POD-8), and it was observed that raised abdominal pressure above 16 mm hg was considered a sensitive predicting factor for burst abdomen using the traditional Foleys method. The peak incidence was found between POD-4 and POD-6. Meena K et al. [10] reported the mean IAP value in the patients with dehiscence was significantly greater than that of the non-dehiscence group (p=0.000). They came to the conclusion that abdominal wound dehiscence is more likely in peritonitis patients enduring protracted surgery in the presence of risk factors such as hypoproteinemia, anaemia, wound infection, postoperative ileus, and postoperative pulmonary infection. Retrospective evaluations typically point to this increase in IAP as the cause, if not the actual cause, of wound dehiscence. Serial IAP measures on D0, D1, and D2 were linked with burst abdomen in a prospective study by Singla et al. [4]. A strong association between IAP on D0 (p=0.000), D1 (p=0.000), and D2 (p=0.000) and burst abdomen was discovered using the t-test. Therefore, based on our research, it can be concluded that patients who subsequently got IAS had greater IAP on these days, while patients who didn’t acquire IAS had lower IAP on the corresponding days.

Because of the study's limited sample size, it is challenging to generalize the findings to the overall population. However, the study's findings provide the fundamental framework for determining the need for a risk-based strategy for treating patients with abdominal injuries who also have the aforementioned risk factors. Future studies that define criteria and scores for the risk variables will improve our ability to forecast the likelihood of complications and take the necessary precautions to prevent them.

## Conclusions

The presence of intra-abdominal sepsis, anemia, and hypoproteinemia favor a higher prevalence of burst abdomen as preoperative risk factors. Among various pathologies, gastroduodenal perforation was found to be the most common pathology associated with a burst abdomen. In the postoperative period, wound infection was the most frequent factor linked with a burst abdomen, followed by raised intra-abdominal pressure (due to cough and vomiting) and postoperative abdominal distension. Intra-abdominal pressure was measured regularly and discovered to be a sensitive early indicator of wound dehiscence, with peak incidence on POD-6 (intra-abdominal pressure>16 mmHg). In both the treatment and prevention of this disorder, adherence to good methods and serious attempts to reduce the influence of predisposing variables are much more important.
